# La Crosse Virus in *Aedes japonicus japonicus* Mosquitoes in the Appalachian Region, United States

**DOI:** 10.3201/eid2104.140734

**Published:** 2015-04

**Authors:** M. Camille Harris, Eric J. Dotseth, Bryan T. Jackson, Steven D. Zink, Paul E. Marek, Laura D. Kramer, Sally L. Paulson, Dana M. Hawley

**Affiliations:** Virginia Polytechnic Institute and State University , Blacksburg, Virginia, USA (M.C. Harris, B.T. Jackson, P.E. Marek, S.L. Paulson, D.M. Hawley);; West Virginia Department of Health and Human Resources, Charleston, West Virginia, USA (E.J. Dotseth);; New York State Department of Health, Slingerlands, New York, USA (S.D. Zink, L.D. Kramer)

**Keywords:** La Crosse virus, disease vector, Appalachian region, Virginia, West Virginia, United States, Aedes japonicus, mosquitoes, viruses, vector-borne infections

## Abstract

La Crosse virus (LACV), a leading cause of arboviral encephalitis in children in the United States, is emerging in Appalachia. For local arboviral surveillance, mosquitoes were tested. LACV RNA was detected and isolated from *Aedes japonicus* mosquitoes. These invasive mosquitoes may significantly affect LACV range expansion and dynamics.

La Crosse virus (LACV; family *Bunyaviridae*, genus *Orthobunyavirus*), in the California serogroup, is the major cause of arboviral encephalitis among children in the United States (*1*). Since its 1963 discovery in Wisconsin, LACV has been identified in 30 other US states (*2*). These include states within the Appalachian Mountain region (West Virginia, Virginia, Ohio, Tennessee, and North Carolina), which is an emerging focus of LACV (*3*). 

The primary vectors of LACV, *Aedes triseriatus* mosquitoes, are present in southwestern Virginia and West Virginia, but 2 invasive congeners—*Ae. albopictus* and *Ae. japonicus*—have recently emerged (*3*). Both species have been shown to be competent experimental LACV vectors (*4*,*5*). Although LACV has been isolated from *Ae. albopictus* mosquitoes (*6*), previously it had only been detected in the Asian bush mosquito (*Ae. japonicus japonicus*) in Tennessee (*7*). *Ae. japonicus* mosquitoes are mammalophilic container breeders that co-occur with the primary LACV vector (*Ae. triseriatus* mosquitoes). Known to feed on humans (*8*), *Ae. japonicus* mosquitoes are found in woodlands (where this “rural encephalitis” virus is endemic) and urban areas (*9*). 

To ascertain the public health risk that *Ae. japonicus* mosquito vectors represent for LACV transmission, we examined mosquitoes from West Virginia and Virginia for presence of this arbovirus. We report 2 independent isolations of LACV from adult *Ae. japonicus* mosquitoes in southwestern Virginia and 7 field detections of LACV RNA from adults (Virginia and West Virginia) and adults reared from eggs (Virginia). Our findings suggest a potential role of this invasive vector in the ecology of LACV in Appalachia (Figure 1).

**Figure 1:**
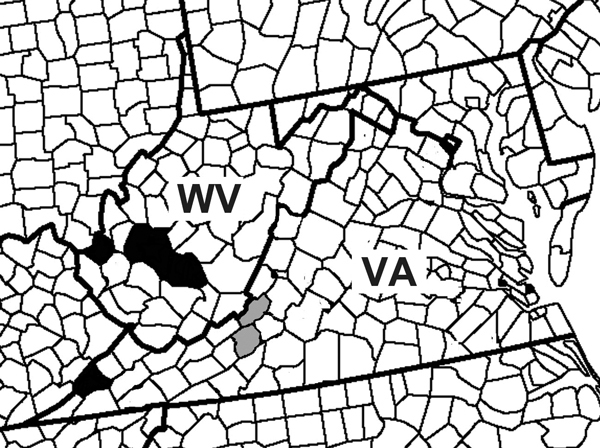
Locations of detection of La Crosse virus (LACV) RNA and virus isolation from *Aedes japonicus* mosquito pools. The red stars represent counties of the *Ae. japonicus* LACV isolates, and the blue stars represent counties of *Ae. japonicus* LACV RNA detection.

## The Study

In 2005, mosquito eggs were collected weekly in Wise County, Virginia, by using ovitraps. The resultant larvae were reared to adults in a Biosafety Level 2 insectary at 24°C, 75% relative humidity, and a photoperiod of 16 hours of light and 8 hours of dark. In 2008 and 2009, adult mosquitoes were collected weekly from infusion-baited gravid traps in Montgomery and Craig Counties, Virginia. Mosquitoes were identified to the species level according to morphology and grouped in pools of <50 individuals according to species, collection location, and collection date. Adults were stored at −80°C until testing. Reverse transcription PCR (RT-PCR) was used for LACV detection in mosquitoes collected in 2005 and 2008. In 2009, mosquito pools were homogenized by using previously described methods for LACV isolation (*6*). Homogenate supernatant (150 μL) was inoculated onto Vero cells, incubated at 37°C, and monitored daily for cytopathic effect. Isolates that showed marked cytopathic effect were harvested and submitted to the Centers for Disease Control and Prevention (CDC) in Fort Collins, Colorado, USA, and the Wadsworth Center in Slingerlands, New York, USA, for quantitative RT-PCR (qRT-PCR).

In 2013, mosquito surveillance was conducted as part of the West Virginia Department of Health and Human Resources Mosquito Surveillance Program. Gravid traps, carbon dioxide–emitting light traps, and BG Sentinel (Biogents AG, Regensburg, Germany) traps baited with octenol lures were used to collect adult mosquitoes weekly from counties with high (Nicholas, Fayette, Raleigh) and low (Kanawha, Jackson, Wood) incidence of LACV among humans as defined (*10*) and on a somewhat regular basis in additional counties. Specimens were pooled by species, county, and collection date for qRT-PCR at the West Virginia Office of Laboratory Services, by use of previously described methods (*11*).

LACV RNA was detected in a pool of *Ae. japonicus* mosquitoes collected as eggs in August 2005 from Wise County, Virginia. LACV was also detected in a pool of *Ae. japonicus* mosquitoes from Montgomery County, Virginia, in July 2008. LACV RNA was detected in 5 separate pools of *Ae. japonicus* mosquitoes collected in West Virginia in 2013, representing 3 counties over a 4-month period. Of 3,529 *Ae. japonicus* mosquitoes collected from Montgomery County in 2009, we isolated LACV from 1 pool (n = 3). In that same year, of 796 *Ae. japonicus* mosquitoes from Craig County tested, LACV was isolated from 1 pool (n = 50) (Table). These isolations were verified by qRT-PCR at CDC (Montgomery County isolate) and the Wadsworth Center (Craig County isolate).

**Table:** Detection of LACV RNA and virus isolations in *Aedes japonicus* mosquito pools from Virginia and West Virginia, USA*

Collection date	County, state	Trap type†	Pool size‡	LACV detection method	C_t_ value	MLE (95% CI)
2005 Aug	Wise, VA	Ovitrap	9	RT-PCR	38.04	8.59 (0.54 –41.00)
2008 Jul	Montgomery, VA	Gravid	22	RT-PCR	37.57	4.51 (0.26–22.00)
2009 Jul	Montgomery, VA	Gravid	3	Isolation, RT-PCR	14.00	0.23 (0.01–1.11)
2009 Jul	Craig, VA	Gravid	50	Isolation, RT-PCR	23.00	1.28 (0.07–6.29)
2013 Jun	Fayette, WV	Multiple adult	36	RT-PCR	37.66	13.41 (5.18–29.14)
2013 Jul	Cabell, WV	Multiple adult	1	RT-PCR	34.72	13.41 (5.18–29.14)
2013 Aug	Fayette, WV	Multiple adult	15	RT-PCR	37.35	13.41 (5.18–29.14)
2013 Aug	Fayette, WV	Multiple adult	2	RT-PCR	34.64	13.41 (5.18–29.14)
2013 Sep	Kanawha, WV	Multiple adult	1	RT-PCR	37.43	13.41 (5.18–29.14)

*C_t_, cycle threshold; LACV, La Crosse virus; MLE, maximum-likelihood estimate of the proportion of infected mosquitoes; RT-PCR, reverse transcription PCR. †All mosquitoes were collected from the field as adults except when ovitraps were used to collect eggs. Adults were reared from the field-collected eggs before testing for arboviruses. Multiple adult traps were gravid traps, carbon dioxide–emitting light traps, and BG Sentinel (Biogents AG, Regensburg, Germany) traps baited with octenol lures. ‡Pool size indicates no. adult mosquitoes tested for LACV.

**Figure 2:**
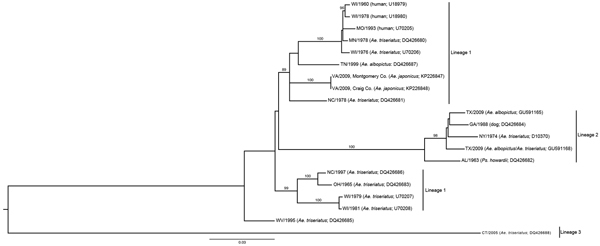
Phylogeny of La Crosse virus (LACV) based on the medium (M) segment of the viral polyprotein gene. State of isolate origin, isolation year, mosquito, or vertebrate isolate source and the National Center for Biotechnology Information (NCBI) accession numbers are listed for each isolate within the tree. The scale bar represents the number of nucleotide substitutions per site. LACV historical lineages are identified by vertical bars. The 2009 isolates from Virginia (NCBI accession nos. KP226847, KP226848) group with lineage 1 viruses. *Ae*., *Aedes*; *Ps*., *Psorophora*.

Nucleotide sequencing and a BLAST (http://blast.ncbi.nlm.nih.gov//Blast.cgi) query were performed on the amplified cDNA from both isolates. The LACV medium segment was used to infer phylogeny (Figure 2). Coding sequences were aligned in Mesquite version 2.75 with Opalescent version 2.10 (*12*,*13*). Phylogenetic trees for the polyprotein genes were estimated by using a maximum likelihood–based method and assuming a general time-reversible (GTR) model with gamma-distributed rate heterogeneity of nucleotide substitution GTR + Γ in RAxML version 8.0.0 (*14*). Support values for each clade were generated in RAxML by using 1,000 rapid bootstrap replicates. The Virginia 2009 isolates were within the previously described lineage I, which contains the reference LACV strain isolated from the brain tissue of a child in Wisconsin (National Center for Biotechnology Information accession no. U18979) (*15*). The 2 isolates from mosquitoes collected in Virginia in 2009 differed by only 1 bp.

## Conclusions

The isolation of LACV from field-collected *Ae. japonicus* mosquitoes, and particularly from mosquitoes collected as eggs, is highly significant because of the pervasiveness of this species in the United States. The large number of LACV detections in this invasive species highlights the need for LACV mosquito surveillance and control efforts to include *Ae. japonicus* in addition to *Ae. triseriatus* mosquito populations. Most (3/5) detections of LACV in West Virginia were in mosquitoes from Fayette County, where incidence of LACV among humans is high, suggesting that *Ae. japonicus* mosquitoes may play a major role in transmission of LACV to humans. Our detection of LACV in *Ae. japonicus* mosquitoes from field-collected eggs in 2005 and the ability of LACV to be transmitted transovarially in *Ae. triseriatus* mosquitoes suggests that future research should examine the possibility of vertical transmission of LACV in invasive *Ae. japonicus* mosquitoes. In states east of the Mississippi River, where *Ae. japonicus* mosquitoes (*9*) and LACV (*2*) co-exist, this mosquito may play a major role in the maintenance, transmission, and range expansion of LACV.
